# Activation of Wnt/β-Catenin Signaling Increases Insulin Sensitivity through a Reciprocal Regulation of Wnt10b and SREBP-1c in Skeletal Muscle Cells

**DOI:** 10.1371/journal.pone.0008509

**Published:** 2009-12-30

**Authors:** Mounira Abiola, Maryline Favier, Eleni Christodoulou-Vafeiadou, Anne-Lise Pichard, Isabelle Martelly, Isabelle Guillet-Deniau

**Affiliations:** 1 Genetics and Development Department, Institut Cochin, Université Paris Descartes, CNRS UMR 8104, Paris, France; 2 Inserm, U567, Paris, France; 3 CRRET, CNRS UMR 7149, Université Paris 12, Créteil, France; 4 UPMC Paris 6, Paris, France; University of Las Palmas de Gran Canaria, Spain

## Abstract

**Background:**

Intramyocellular lipid accumulation is strongly related to insulin resistance in humans, and we have shown that high glucose concentration induced de novo lipogenesis and insulin resistance in murin muscle cells. Alterations in Wnt signaling impact the balance between myogenic and adipogenic programs in myoblasts, partly due to the decrease of Wnt10b protein. As recent studies point towards a role for Wnt signaling in the pathogenesis of type 2 diabetes, we hypothesized that activation of Wnt signaling could play a crucial role in muscle insulin sensitivity.

**Methodology/Principal Findings:**

Here we demonstrate that SREBP-1c and Wnt10b display inverse expression patterns during muscle ontogenesis and regeneration, as well as during satellite cells differentiation. The Wnt/β-catenin pathway was reactivated in contracting myotubes using siRNA mediated SREBP-1 knockdown, Wnt10b over-expression or inhibition of GSK-3β, whereas Wnt signaling was inhibited in myoblasts through silencing of Wnt10b. SREBP-1 knockdown was sufficient to induce Wnt10b protein expression in contracting myotubes and to activate the Wnt/β-catenin pathway. Conversely, silencing Wnt10b in myoblasts induced SREBP-1c protein expression, suggesting a reciprocal regulation. Stimulation of the Wnt/β-catenin pathway i) drastically decreased SREBP-1c protein and intramyocellular lipid deposition in myotubes; ii) increased basal glucose transport in both insulin-sensitive and insulin-resistant myotubes through a differential activation of Akt and AMPK pathways; iii) restored insulin sensitivity in insulin-resistant myotubes.

**Conclusions/Significance:**

We conclude that activation of Wnt/β-catenin signaling in skeletal muscle cells improved insulin sensitivity by i) decreasing intramyocellular lipid deposition through downregulation of SREBP-1c; ii) increasing insulin effects through a differential activation of the Akt/PKB and AMPK pathways; iii) inhibiting the MAPK pathway. A crosstalk between these pathways and Wnt/β-catenin signaling in skeletal muscle opens the exciting possibility that organ-selective modulation of Wnt signaling might become an attractive therapeutic target in regenerative medicine and to treat obese and diabetic populations.

## Introduction

The first suggestion for a role of Wnt signaling in the pathogenesis of type 2 diabetes came from a study which reported a single nucleotide polymorphism locus in the Wnt5b gene that confered susceptibility to type 2 diabetes in a Japonese population [1]. More recently, variants of the transcription factor TCF7L2, a component of the Wnt/β-catenin pathway, were shown to be involved in β-cell dysfunction and the etiology of type 2 diabetes [2]. In addition, a link between cellular glucose sensing and the Wnt/β-catenin pathway was recently reported in macrophages [3], indicating that this pathway could be inappropriately activated in diabetic hyperglycemic or obese subjects. These results strongly suggest that Wnt signaling could be involved in the regulation of glucose homeostasis in different organs, particularly in insulin-responsive tissues such as skeletal muscle.

### The Wnt/β-Catenin Signaling Pathway

A central feature of the canonical Wnt/β-catenin pathway is the regulation of cytosolic β-catenin protein levels via a destruction complex containing glycogen synthase kinase-3β (GSK-3β), adenomatous polyposis coli (APC) and axin. In the absence of Wnt signals, β-catenin is targeted for ubiquitin-mediated degradation [4]. Binding of Wnt ligands to a Frizzled/LRP receptor complex leads to the inactivation of GSK-3β and accumulation of cytosolic β-catenin. Then β-catenin translocates into the nucleus where it binds to TCF/LEF transcription factors to activate transcription of Wnt-responsive genes involved in cell proliferation (cyclin D1, myf5) and differentiation [5], [6]. Wnt signaling also plays a key role in adult tissues homeostasis by determining differentiating cell fate and maintaining stem cell pluripotency [7].

### Shift in Lipid Metabolism and Muscle Insulin Resistance

Several tissues, including skeletal muscle, display with ageing an adverse shift in lipid metabolism which contributes to insulin resistance and type 2 diabetes [8]. Insulin resistance has been linked to the accumulation of intramyocellular lipids in skeletal muscle of diabetic patients [9], [10] in relation with the lipogenic transcription factor SREBP-1c which mediates insulin's actions on hepatic [11], [12], [13] and skeletal muscle gene expression in humans [14] and rodents [15]. We have shown that adenoviral delivery of SREBP-1 gene to cultured rat muscle satellite cells resulted in a gene expression profile that would suppress fat oxidation and promote intramyocellular lipid accumulation [15], suggesting that SREBP-1c plays a role in the development and/or maintenance of skeletal muscle insulin resistance.

### Role of Wnt Signaling in the Balance Adipogenesis/Myogenesis

Down-regulation of Wnt signaling may alter myoblastic differentiation potential as a function of age, as it controls the balance between myogenic and adipogenic potential in myoblasts [16], suggesting that endogenous Wnt signaling inhibits adipogenesis [17]. Although myoblast determination and differentiation are regulated by myogenic transcription factors (myf5, myoD, myogenin) [18], their activity can be overcome in vitro so that myoblasts can be induced to transdifferentiate into adipocyte-like cells by treatment with thiazolidinediones, potent activators of PPAR-γ [19], [20], or high glucose concentration [21]. These results strongly suggest that skeletal muscle satellite cells are able to enter an adipogenic program under particular pathophysiological conditions.

We have shown previously that high glucose concentration up-regulated SREBP-1c in cultured muscle satellite cells, leading to de novo lipogenesis and insulin resistance [15]. Here we show a reciprocal regulation between SREBP-1c and Wnt10b mRNA and protein expression in muscle cells, in relation with intramyocellular lipid deposition and insulin resistance. Surprisingly, stimulation of the Wnt/β-catenin pathway through SREBP-1c knockdown, GSK-3β inhibition or Wnt10b over-expression prevented intramyocellular lipid synthesis, redirected myotubes toward a myogenic phenotype and restored insulin sensitivity in insulin-resistant myotubes through a molecular mechanism involving a differential activation of Akt/PKB and AMPK pathways.

## Results

### SREBP-1c and Wnt10b Proteins Show an Inverse Expression Pattern In Vivo

**Figure 1:**
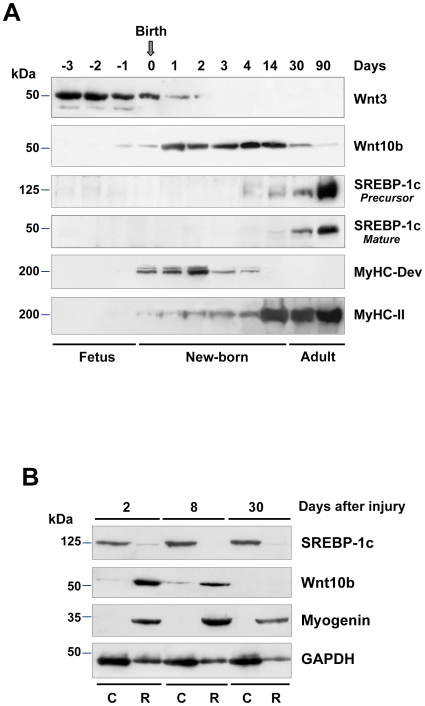
Differential expression of SREBP-1c and Wnt proteins during skeletal muscle ontogenesis and regeneration. (**A**) Western blot analysis showing inverse expression patterns between Wnt10b and SREBP-1c during ontogenesis. The developmental stages are underlined using antibodies against developmental (MyHC-Dev) and fast (MyHC-2) myosin heavy chains. (**B**) Western blot analysis of Wnt10b and SREBP-1c protein levels in regenerating (R) adult EDL muscles at 2, 8, and 30 days after crush injury as compared to contralateral control (C) EDL. The down-regulation of SREBP-1c was concomitant with the up-regulation of Wnt10b throughout regeneration. The blots are representative of 3 independent experiments.

Figure 1A represents the evolution of SREBP-1c and Wnt proteins that are expressed during skeletal muscle ontogenesis. Wnt3 protein was strongly expressed in hind limb muscles of rat fetuses, but was rapidly down-regulated after birth, whereas Wnt5a and Wnt7 were not detected (result not shown). In contrast, Wnt10b protein was barely detectable in fetal muscles, but was strongly up-regulated after birth and remained elevated in newborn muscles throughout suckling. Then it decreased drastically after weaning to become quite undetectable in adult muscles. Conversely, SREBP-1c protein was not expressed in muscles from birth to weaning while Wnt10b protein level was elevated. After weaning, SREBP-1c precursor and mature forms increased by 5-fold when muscle growth was over, as shown by a strong expression of the fast Myosin Heavy Chain-2 protein (MyHC-2). These results suggest that Wnt10b protein could be only expressed in myoblasts that are involved in postnatal skeletal muscle growth, a period during which the lipogenic factor SREBP-1c is not detected.

To challenge this hypothesis, we assessed the differential expression of Wnt10b and SREBP-1c proteins during skeletal muscle regeneration in adult EDL muscles. EDL muscle from the left hind limb was crushed using tweezers as previously described [22] and the non-injured contralateral EDL was taken as a control. Two days after crush injury, Wnt10b protein levels increased dramatically and remained elevated until 8 days following the injury, then became undetectable after 30 days when regeneration was over. Myogenin was transiently up-regulated during the regeneration process, whereas SREBP-1c was completely down-regulated (Figure 1B). These findings suggest that Wnt10b might activate a signaling pathway that prevents the expression of lipogenic factors during skeletal muscle growth and regeneration.

### SREBP-1c and Wnt10b Show an Inverse Expression Pattern in Cultured Satellite Cells

**Figure 2:**
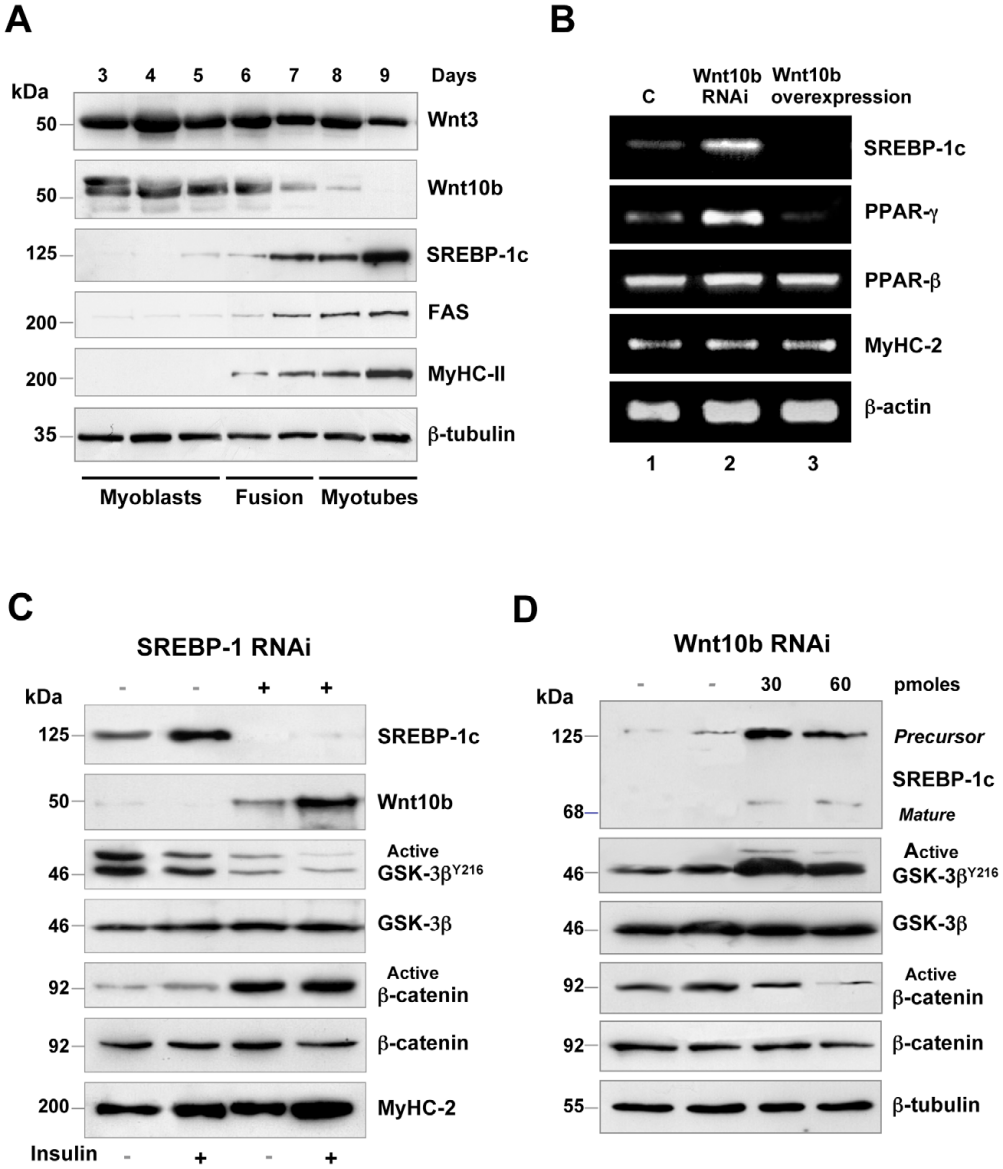
Wnt10b and SREBP-1c are also inversely expressed in cultured satellite cells. (**A**) Western blot analysis showing an inverse expression pattern between Wnt10b and SREBP-1c proteins according to the differentiation stage. In contrast, Wnt3 remained almost unchanged throughout differentiation. SREBP-1c induced the up-regulation of the lipogenic enzyme FAS in myotubes. (**B**) Wnt10b knockdown was sufficient to up-regulate SREBP-1c and PPARγ mRNAs, whereas Wnt10b over-expression down-regulated their expression. RT-PCR was performed on myoblasts transfected with a scrambled siRNA (lane 1), a pool of 3 Wnt10b siRNAs (lane 2), or a plasmid encoding the mouse Wnt10 cDNA (lane 3) as described in Material and Methods. (**C**) SREBP-1 knockdown stimulated Wnt signaling in contracting myotubes. Myotubes were transfected with SREBP-1 siRNAs or a scrambled siRNA, then treated or not with 10 nM insulin for 24 hours. SREBP-1 knockdown was sufficient to induce Wnt10b protein expression in myotubes, particularly in the presence of insulin, and to activate the Wnt/β-catenin pathway, as shown by GSK-3β and β-catenin activities. (**D**) Wnt10b knockdown induced SREBP-1c protein expression in myoblasts. Myoblasts were transfected with 30 pmoles or 60 pmoles of a pool of 3 Wnt10b siRNAs, or with a scrambled siRNA as a control. Silencing Wnt10b was sufficient to induce SREBP-1c protein expression in myoblasts through the inhibition of Wnt/β-catenin signaling. The blots are representative of 3 independent experiments.

As muscle growth and regeneration occur through the activation of satellite cells, we studied the expression of Wnt10b protein in primary cultures of satellite cells isolated from hind limb muscles. Myoblasts were allowed to proliferate for 5 days, then fusion was induced and spontaneously contracting myotubes were obtained at day 9. Wnt10b protein was strongly expressed in proliferating myoblasts, then decreased during cell fusion and was no longer detectable in contracting myotubes (Figure 2A), whereas Wnt3 protein expression remained almost unchanged. In contrast, Wnt10b down-regulation was concomitant with the up-regulation of SREBP-1c and fatty acid synthase (FAS) proteins, showing that lipogenesis was active in contracting myotubes (Figure 2A). These results demonstrated that Wnt10b and SREBP-1c proteins adopted inverse expression patterns according to the differentiation stage of myogenic cells.

### Wnt10b Silencing Increases SREBP-1c mRNA Expression in Myoblasts

To determine whether Wnt10b knockdown could induce SREBP-1c expression, myoblasts were transfected with a mix of 3 specific Wnt10b siRNAs or with a control scrambled siRNA. In parallel, myotubes were transfected with a vector containing mouse Wnt10b cDNA. Total RNA was extracted 48 hours after transfection and semi-quantitative PCR was performed. Wnt10b knockdown was sufficient to induce SREBP-1c mRNA expression in myoblasts, whereas Wnt10b over-expression drastically decreased SREBP-1c mRNA expression in myotubes (Figure 2B). Furthermore, Wnt10b knockdown increased by 3-fold PPAR-γ mRNA expression in myoblasts, whereas Wnt10b over-expression decreased PPAR-γ mRNA in myotubes. In contrast, PPAR-β and MyHC-2 mRNAs were unaffected. These results show that Wnt10b decreased SREBP-1c and PPAR-γ expression at the transcriptional level.

### SREBP-1c Silencing Activates the Wnt/β-Catenin Pathway in Contracting Myotubes

In order to determine whether SREBP-1c could inhibit Wnt10b expression, myotubes were transfected with a SREBP-1 siRNA duplex or with a control scrambled siRNA, and total proteins were extracted 48 hours later. SREBP-1c protein was hardly detectable in siRNA-transfected myotubes as compared with control myotubes, indicating a knockdown efficiency of more than 90%. Gene silencing was successful even in the presence of 10 nM insulin, a potent activator of SREBP-1c transcription. Surprisingly, SREBP-1c knockdown was sufficient to induce Wnt10b protein expression in contracting myotubes, particularly in the presence of insulin (Figure 2C). To determine whether SREBP-1c knockdown stimulated Wnt/β-catenin signaling, the activity of GSK-3β was checked using an antibody raised against phosphorylated GSK-3β^Y216^ which is active when phosphorylated, but is inactive when dephosphorylated in response to Wnt/β-catenin signaling [23]. GSK-3β activity decreased by 3-fold in SREBP-1c knocked-down myotubes. Furthermore, the dephosphorylated active form of β-catenin was increased in these cells, while total GSK-3β and total β-catenin remained unchanged. MyHC-2 level showed that myotubes remained terminally differentiated after SREBP-1c knockdown (Figure 2C). Taken together, these results suggest that SREBP-1c could be involved in the down-regulation of Wnt signaling which is observed in contracting myotubes.

### Wnt10b Silencing Induces SREBP-1c Protein Expression in Myoblasts

We wanted to determine whether Wnt10b knockdown could conversely induce the expression of SREBP-1c protein in myoblasts. Total protein was extracted 48 hours after transfection of myoblasts with a mix of 3 specific Wnt10b siRNAs or with a control scrambled siRNA. Wnt10b knockdown was sufficient to induce 24 hours later SREBP-1c protein expression in myoblasts. The concomitant 2 to 3-fold increase in GSK-3β^Y216^ phosphorylation led to the down-regulation of active β-catenin in transfected myoblasts, showing an inhibition of the Wnt/β-catenin pathway (Figure 2D).

### Forced-Expression of Wnt10b Down-Regulates SREBP-1c in Contracting Myotubes

**Figure 3:**
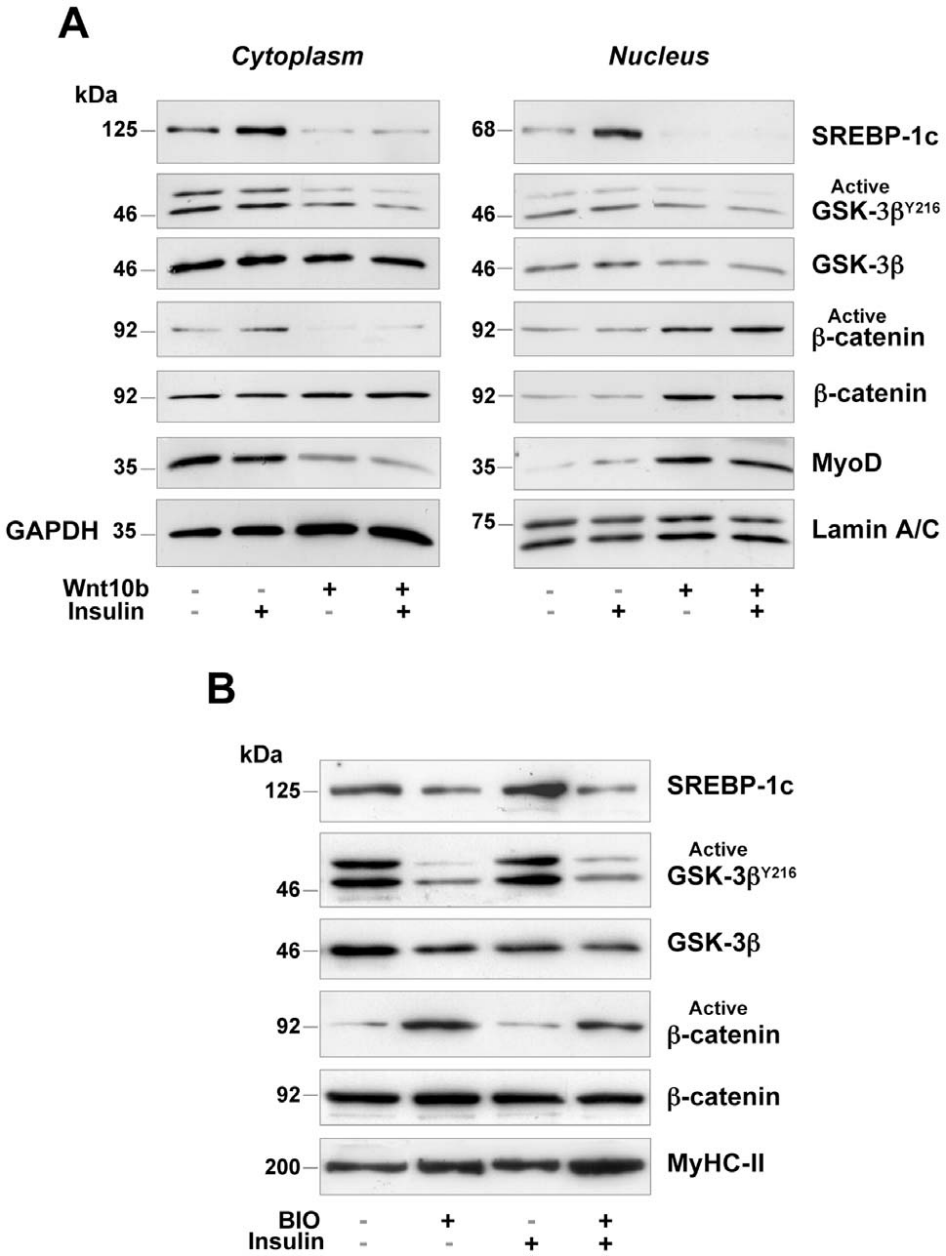
Activation of Wnt signaling in contracting myotubes. (**A**) Over-expression of Wnt10b cDNA down-regulated SREBP-1c protein. Myotubes were transfected with a plasmid encoding the mouse Wnt10b cDNA, then treated or not with 10 nM insulin for 24 hours. Western blot analysis of cytoplasmic (left panel) and nuclear (right panel) protein extracts showing the down-regulation of precursor and mature forms of SREBP-1c following the activation of the Wnt/β-catenin pathway. Wnt10b over-expression induced the nuclear accumulation of active β-catenin and MyoD. Blots were normalized using antibodies raised against the cytoplasmic protein GAPDH or the nuclear protein Lamin A/C. (**B**) Myotubes were submitted to a 48 hour-treatment with 1 µM BIO, then 10 nM insulin was added for 24 hours. BIO-mediated activation of the Wnt/β-catenin pathway induced SREBP-1c down-regulation, even in the presence of insulin.

In order to determine whether the over-expression of Wnt10b could be sufficient to down-regulate SREBP-1c in myotubes, cells were transfected with a plasmid encoding mouse Wnt10b cDNA, and cytosolic and nuclear proteins were extracted 48 hours later. Wnt10b over-expression was sufficient to totally abrogate the expression of the cytoplasmic-precursor and nuclear-active forms of SREBP-1c, even in the presence of insulin (Figure 3A). As expected, Wnt10b over-expression decreased GSK-3β activity by dephosphorylating Y^216^ and induced the nuclear translocation of active β-catenin, which led to the up-regulation of MyoD in the nucleus. These results demonstrate that the reactivation of the Wnt/β-catenin pathway down-regulated the lipogenic factor SREBP-1c, but also stimulated the myogenic pathway in contracting myotubes. Altogether, our data suggest that the reciprocal regulation between SREBP-1c and Wnt10b resulted from a crosstalk between Wnt/β-catenin signaling and the transcription factor SREBP-1c.

### Activation of the Wnt/β-Catenin Pathway Down-Regulates SREBP-1c in Myotubes

Myotubes were treated for 48 hours with 6-BromoIndirubin-3′-Oxime (BIO), a selective inhibitor of GSK-3 activity that activates the Wnt/β-catenin pathway [24]. BIO decreased GSK-3β activity by preventing Y^216^ phosphorylation (Figure 3B), but had no effect on S^9^ phosphorylation. As expected, the inhibition of GSK-3β up-regulated the active form of β-catenin, resulting in SREBP-1c down-regulation. These results show that reactivation of the Wnt/β-catenin pathway whatever the technique used (SREBP-1c knockdown, Wnt10b over-expression or selective GSK-3β inhibition) drastically diminished the lipogenic factor SREBP-1c in contracting myotubes.

### BIO Abolishes Glucose-Induced Intramyocellular Lipid Accumulation in Myotubes

**Figure 4:**
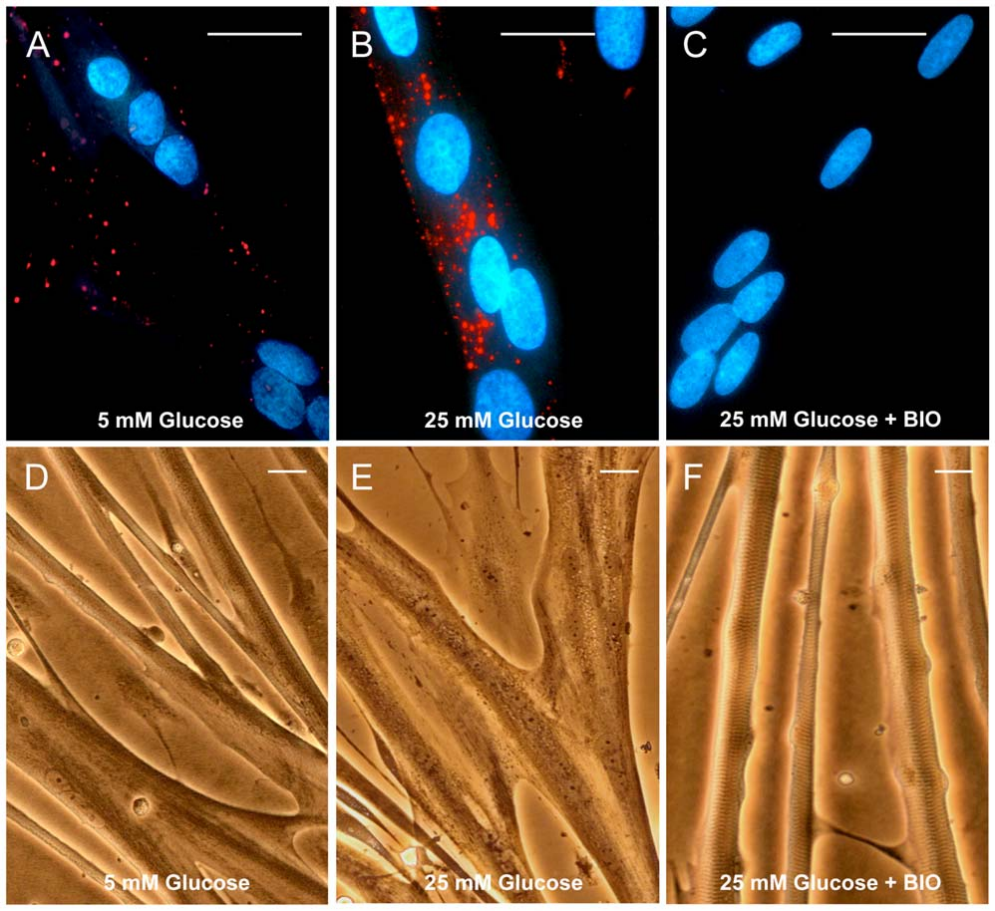
Effect of BIO on intramyocellular lipid accumulation. Oil red O staining of intramyocellular lipids in myotubes cultured in 5 mM glucose (**A**) 25 mM glucose (**B**) or 25 mM glucose in the presence of 1 µM BIO for 5 days (**C**). Phase-contrast microphotographs of the same myotubes cultured in 5 mM glucose (**D**), or 25 mM glucose in the absence (**E**) or presence (**F**) of BIO. BIO totally abolished intramyocellular lipid deposition. Scale bar 20 µm.

In myotubes cultured under physiological glucose concentration (5 mM), few lipid droplets were detected using Oil Red O staining (Figure 4A), whereas high glucose concentration (25 mM) drastically increased SREBP-1c-mediated de novo lipogenesis (Figure 4B), confirming what we have previously reported [25]. In contrast, no intramyocellular lipids were detected in myotubes treated with 1 µM BIO (Figure 4C). In addition, BIO was able to induce the formation of terminally-differentiated contracting myotubes, as the contractile apparatus was visible and nuclei appeared in a peripheral position (Figure 4F). Thus, activation of Wnt/β-catenin signaling redirected muscle cells toward the myogenic pathway.

### Effect of Wnt Signaling on 2-Deoxyglucose Transport in Contracting Myotubes

**Figure 5:**
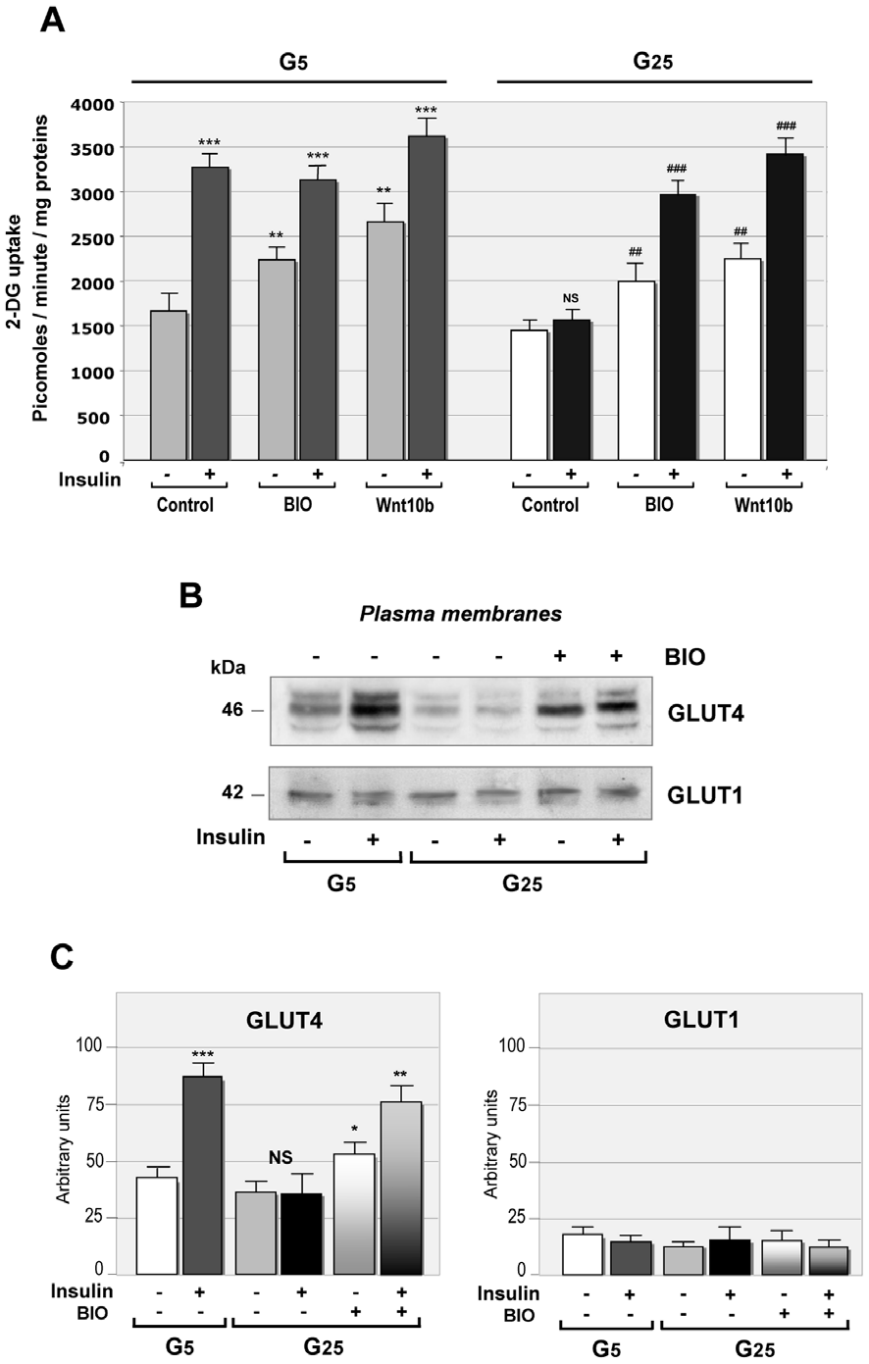
Effect of Wnt signaling on glucose transport in contracting myotubes. (**A**) Myotubes cultured in 5 mM glucose (G5) or in 25 mM glucose (G25) for 48 hours were transfected with a mouse Wnt10b cDNA or treated with 1 µM BIO. 2-deoxyglucose (2-DG) uptake was then measured in the presence or absence of 10 nM insulin for 30 minutes as described in Material and Methods. Data are expressed as mean±SE from 5 independent experiments performed in triplicate. Significant difference from G5, (***) p<0.0001; (**) p<0.02; Significant difference from G25, (###) p<0.0001; (##) p<0.001. (**B**) BIO induced GLUT4 translocation to the plasma membrane. Myotubes were cultured in 5 mM (G5) or 25 mM glucose (G25) for 48 hours in the presence or absence of 1 µM BIO. Myotubes were treated or not with 10 nM insulin for 30 minutes, then plasma membranes were isolated. Western blot analysis showed that insulin and BIO induced GLUT4 translocation to the plasma membrane, whereas GLUT1 was unaffected. (**C**) Quantification of GLUT4 and GLUT1 translocation. Data are expressed as mean±SE from 4 independent experiments. Significant difference from G5, (***) p<0.0001; (**) p<0.01; (*) p<0.05.

As glucose transport reflects insulin sensitivity in skeletal muscle, H^3^-2-Deoxyglucose (2-DG) uptake was measured in contracting myotubes. Myotubes cultured under physiological glucose concentration (G5) showed high insulin sensitivity, as 10 nM insulin induced within 30 minutes a 2-fold increase in 2-deoxyglucose uptake. Surprisingly, BIO treatment or Wnt10b over-expression increased basal 2-deoxyglucose uptake by 30% and 40% respectively, whereas uptake in the presence of BIO and insulin was similar to insulin-stimulated uptake (Figure 5A, left panel). In contrast, myotubes cultured for 48 hours in high glucose concentration (G25) presented a drastic insulin-resistance, as insulin was unable to stimulate 2-deoxyglucose uptake. However, BIO or Wnt10b over-expression increased basal glucose uptake by 20% and 40%, respectively, and restored insulin sensitivity in these myotubes (Figure 5A, right panel). Thus, the activation of Wnt signaling had no effect on insulin-stimulated glucose uptake in insulin-sensitive conditions, but increased glucose transport independent of insulin and restored insulin sensitivity in insulin-resistant myotubes.

### BIO Induces GLUT4 Translocation

In skeletal muscle, glucose transport is carried out by the insulin-sensitive glucose transporter GLUT4. In myotubes cultured in G5, insulin induced GLUT4 translocation to the plasma membrane within 30 minutes (Figure 5B). In contrast, insulin did not induce GLUT4 translocation in myotubes cultured in G25, confirming a strong insulin resistance. Surprisingly, BIO per se induced GLUT4 translocation to the plasma membrane in G25-cultured myotubes, and insulin had an additive effect. This effect was specific for GLUT4, as GLUT1 was unaffected (Figure 5B, Figure 5C). This experiments showed that activation of Wnt/β-catenin signaling induced GLUT4 translocation to the plasma membrane through an insulin independent pathway.

### Effect of BIO on GSK-3β Activity

**Figure 6:**
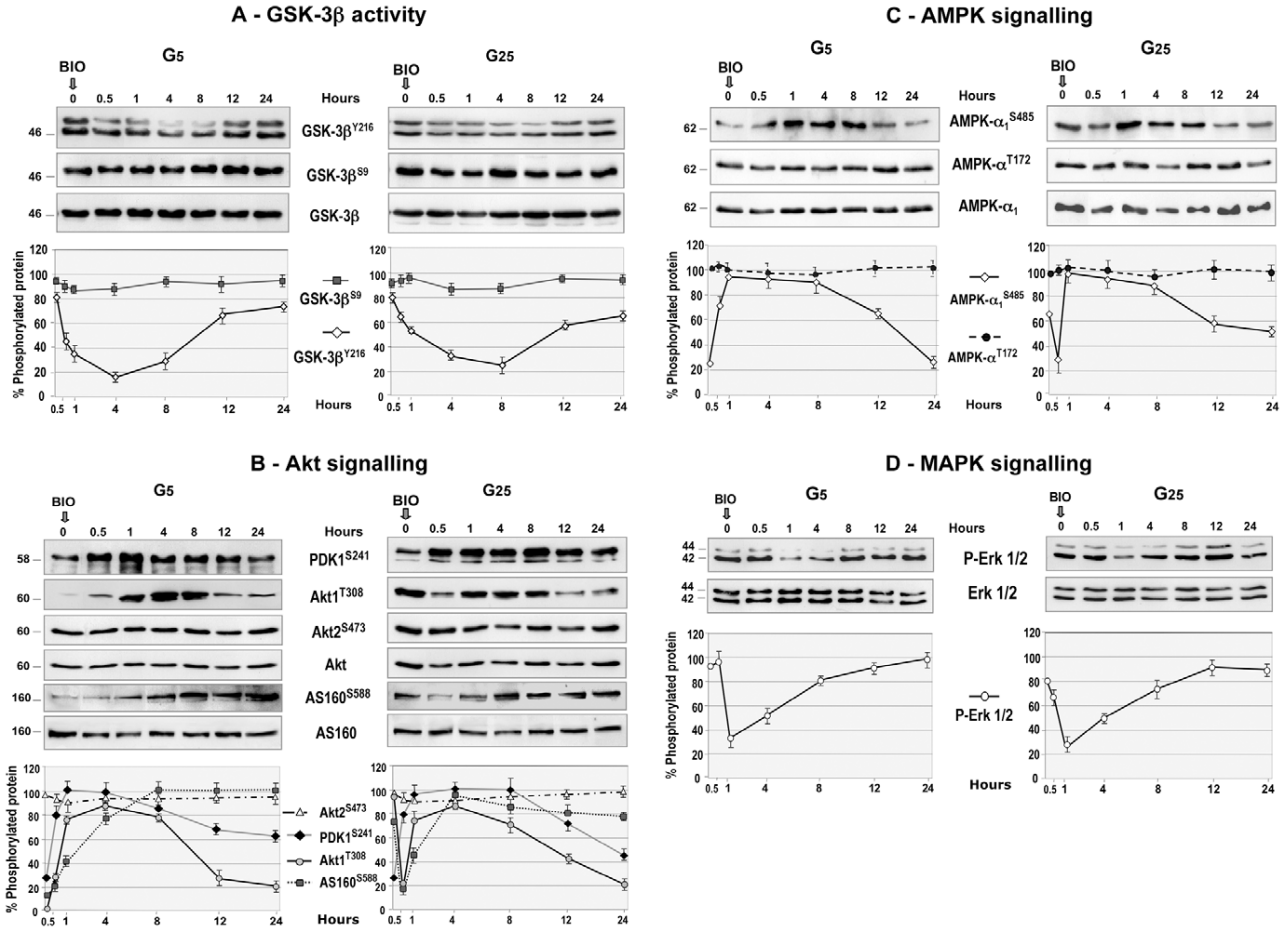
Time-course of BIO effects on GSK-3β activity, Akt, AMPK and MAPK signaling in contracting myotubes. Myotubes were cultured in 5 mM (G5) or 25 mM (G25) glucose concentration for 48 hours, then 1 µM BIO was added. Upper panels show western blots whereas lower panels show quantifications of 3 to 5 independent experiments. (**A**) BIO decreased within 30 minutes GSK-3β^Y216^ phosphorylation in myotubes cultured either in G5 or G25, but had no effect on GSK-3β^S9^ phosphorylation. (**B**) BIO induced PDK1^S241^, Akt1^T308^ and AS160^S588^ phosphorylations, but had no effect on Akt2^S473^ phosphorylation in myotubes cultured either in G5 or G25. (**C**) BIO increased AMPK-α1^S485^ phosphorylation, but had no effect on AMPK-α^T172^ phosphorylation in myotubes cultured in G5 (left panels). In contrast, BIO had a biphasic effect in myotubes cultured in G25: AMPK-α1^S485^ phosphorylation was decreased to a basal level within 30 minutes, then increased with a time-course similar to that observed in myotubes cultured in G5 (right panels). (**D**) BIO decreased Erk1/2 phosphorylation with a similar time-course in myotubes cultured either in G5 or G25. Data are expressed as mean±SE from 3 independent experiments.

In order to decipher the signaling pathways involved in BIO effects, we performed time-course experiments in myotubes cultured in G5 or G25. Within 30 minutes, BIO decreased GSK-3β activity, as assessed by Y^216^ phosphorylation, and maximal inhibition (70% and 60% in myotubes cultured in G5 and G25 respectively) was observed after about 4 hours, then remained for 12 hours in both cases. In contrast, BIO had no significant effect on GSK-3β^S9^ phosphorylation (Figure 6A).

### Effect of BIO on Akt Signaling

Akt/PKB is a protein kinase activated by insulin and various growth factors through pathways involving PI3 kinase. Akt1 is activated by activation-loop phosphorylation at T^308^ by the pyruvate dehydrogenase kinase 1 (PDK1) [26], and Akt2 by phosphorylation within the carboxy terminus at S^473^ by mTor [27]. Our data show in myotubes cultured in G5 that BIO increased PDK1^S241^ autophosphorylation within 30 minutes, which induced Akt1^T308^ phosphorylation for 8 hours, but had no effect on Akt2^S473^ phosphorylation. Finally the Akt substrate AS160 (a Rab-GAPase involved in GLUT4 translocation) was activated by phosphorylation on S^588^ for at least 24 hours (Figure 6B, left panel). In contrast, Akt1^T308^ and AS160^S588^ were already highly phosphorylated in myotubes cultured in G25. Surprisingly, the first effect of BIO was to bring the phosphorylation state back to a basal level within 30 min. Then BIO up-regulated Akt1^T308^ and AS160^S588^ phosphorylations that remained elevated until 8 hours after BIO addition (Figure 6B, right panel). These results could explain how BIO induced GLUT4 translocation and increased glucose uptake in myotubes that were either sensitive or resistant to insulin action.

### Effect of BIO on AMP Kinase Signaling

AMPK promotes GLUT4 translocation and glucose uptake in skeletal muscle by a signaling cascade independent of the classical insulin-PI3K-Akt pathway [28]. Recently, Jensen et al [29] showed that twitch-contraction stimulated glucose uptake through the activation of AMPK-α1 (but not AMPK-α2) independently of AS160 phosphorylation in mouse skeletal muscle. Figure 6C shows that BIO induced rapidly the phosphorylation of AMPK-α1 on S^485^, but had no effect on T^172^ phosphorylation in myotubes cultured in G5. In myotubes cultured in G25, AMPK-α1^S485^ phosphorylation was 3-fold higher than in myotubes cultured in G5. Addition of BIO down-regulated AMPK-α1^S485^ phosphorylation to a basal level within 30 minutes, then AMPK-α1^S485^ was phosphorylated according to a time-course similar to the one observed in myotubes cultured in G5 (Figure 6C).

### Effect of BIO on MAP Kinase Signaling

As the MAP kinase (MAPK) pathway is involved in insulin signal transduction, we wonder whether BIO, which was designed as a specific GSK-3β inhibitor [30], could indirectly affect this pathway. Figure 6D shows that the rapid (30 minutes) inhibition of GSK-3β inhibited Erk1/2 phosphorylation after 1 hour, this effect remaining for almost 12 hours in myotubes cultured in G5 or G25. Taken together these results show that BIO induced, as a downstream consequence of GSK-3β inactivation, the inhibition of the MAP kinase pathway and the activation of Akt/PKB and AMPK pathways in skeletal muscle cells.

### Comparison between Insulin and BIO Signaling in Contracting Myotubes

#### GSK-3β activity

**Figure 7:**
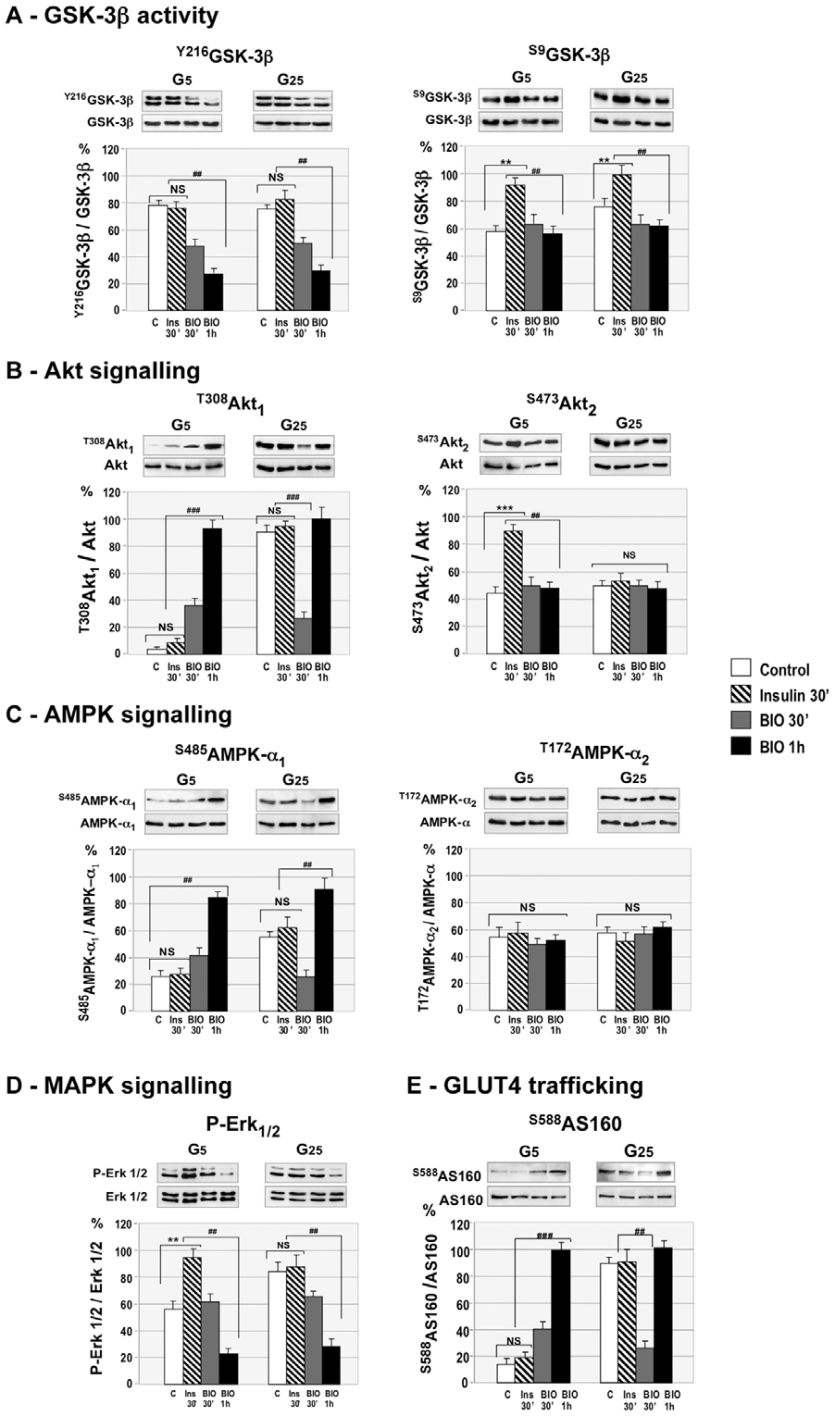
Comparison between insulin and BIO effects on intracellular signaling. Myotubes cultured in 5 mM glucose (G5) or 25 mM glucose (G25) concentration were treated with 10 nM insulin for 30 minutes, or with 1 µM BIO for 30 and 60 minutes. (**A**) Insulin increased GSK-3β^S9^ phosphorylation in myotubes cultured in G5 or G25, whereas BIO had no effect. In contrast, BIO decreased GSK-3β^Y216^ phosphorylation, whereas insulin had no effect. (**B**) Insulin increased Akt2^S473^ phosphorylation in myotubes cultured in G5, whereas myotubes cultured in G25 were resistant to insulin. BIO increased Akt1^T308^ phosphorylation in myotubes cultured in G5, and had a biphasic effect in myotubes cultured in G25. (**C**) BIO stimulated AMPK-α1^S485^ but not AMPK-α2^T172^ phosphorylation whatever the glucose concentration, whereas insulin had no effect on AMPK phosphorylation. (**D**) In contrast to BIO, insulin failed to increase AS160^S588^ phosphorylation. BIO showed a biphasic effect in myotubes cultured in G25. (**E**) Insulin increased Erk1/2 phosphorylation only in myotubes cultured in G5, whereas BIO diminished Erk1/2 phosphorylation in myotubes whatever the glucose concentration. Lower panels show quantifications of 3 independent experiments. Data are expressed as mean±SE. Significant difference between BIO and insulin, (###) p<0.001; (##) p<0.01. Significant difference between control and insulin (***) p<0.001; (**) p<0.01. NS: non significant.

Myotubes cultured either in G5 or G25 were treated for 30 minutes with 10 nM insulin, and for 30 minutes or 1 hour with 1 µM BIO. Quantifications of western-blots show that GSK-3β activity was inhibited in myotubes cultured in low or high glucose concentration through either insulin-induced phosphorylation of S^9^, or BIO-induced dephosphorylation of Y^216^ (Figure 7A).

#### Akt signaling

While insulin stimulated Akt2^S473^ phosphorylation, BIO increased Akt1^T308^ phosphorylation (Figure 7B), leading to AS160^S588^ activation in myotubes cultured in G5 (Figure 7D). In contrast, insulin did not stimulate Akt2^S473^ phosphorylation in myotubes cultured in G25, whereas BIO induced a biphasic response: it first decreased Akt1^T308^ and AS160^S588^ phosphorylations to a basal level within 30 min, then reinduced these phosphorylations (Figures 7B, 7D). These results show that BIO is able to activate the Akt pathway in insulin-resistant myotubes through a way different from the classical insulin/PI3K pathway.

#### AMPK signaling

Insulin had no effect on AMPK signaling in myotubes cultured either in G5 or G25. In contrast, BIO induced AMPK-α1^S485^ phosphorylation whatever the glucose concentration, but had no effect on AMPK-α^T172^ phosphorylation. Thus, BIO stimulated AMPK signaling through AMPK-α1^S485^ phosphorylation (Figure 7C).

#### MAPK signaling

BIO and insulin had opposite effects on MAPK signaling. BIO induced Erk1/2 dephosphorylation within 1 hour in myotubes cultured in G5 or G25, whereas insulin increased Erk1/2 phosphorylation in myotubes cultured in G5, but had no effect in myotubes cultured in G25 (Figure 7D).

Taken together our results show that activation of the Wnt/β-catenin pathway prevented an adipogenic phenotype and improved insulin sensitivity in skeletal muscle cells through a differential stimulation of the Akt and MAPK pathways.

## Discussion

Our previous work demonstrated that high glucose concentration up-regulated SREBP-1c and insulin resistance in myotubes [25]. As Wnt proteins are known to initiate myogenesis [5], [31] and inhibit adipogenic differentiation [32], [33], we hypothesized that Wnt10b normally represses the expression of lipogenic genes such as SREBP-1c in skeletal muscle.

### Wnt/β-Catenin Signaling Overcomes an Adipogenic Program in Muscle

Here we show that Wnt10b was detected as long as SREBP-1c was not present in growing muscles of suckling rats (Figures 1A, 2A). As SREBP-1c is sensitive to the nutritional status in skeletal muscle [34], we cannot rule out a role for poly-unsaturated fatty acids (PUFAs) which are abundant in mouse milk. Conversely, a strong expression of SREBP-1c in adult muscle was concomitant with the loss of Wnt10b. On the contrary, the regeneration process reinduced Wnt10b protein expression and totally down-regulated SREBP-1c in EDL muscle (Figure 1B). This is supported by the observation that muscle regeneration was impaired in the Tibialis Anterior of Wnt10b^−^/^−^ mice, where excessive lipid accumulation occurred within activated satellite cells and regenerating myofibers [35]. Thus, upon satellite cells activation, which started the myogenic program, the lipogenic protein SREBP-1c remained absent. These findings were confirmed in vitro as, whatever the technique used (SREBP-1 knockdown, Wnt10b over-expression or GSK-3β inbibition through BIO), activation of the Wnt/β-catenin pathway decreased SREBP-1c mRNA and protein levels in contracting myotubes. Conversely, silencing Wnt10b was sufficient to increase SREBP-1c mRNA and protein as well as the adipogenic phenotype of myoblasts, as shown by the up-regulation of PPAR-γ mRNAs.

These results suggest that activation of Wnt signaling overcame an adipogenic program in muscle satellite cells. Such findings could be very important in the general context of muscle development, but also in the specific context of obesity and type 2 diabetes. In these pathologies, skeletal muscle has been observed to have a reduced oxidative enzyme activity, increased glycolytic activity, and increased lipid content. These metabolic characteristics are related to skeletal muscle insulin resistance and are factors potentially related to muscle fiber type [36], particularly for fast MyHC-expressing fibers [31]. To address this important topic, we are now studying the effects of direct electrotransfection of Wnt10b and other Wnt factors in mouse Tibialis Anterior and Soleus muscles on fiber-type composition, metabolism, intramyocellular lipid content and insulin sensitivity.

### Wnt10b and SREBP-1c Are Mutually Exclusive in Muscle

**Figure 8:**
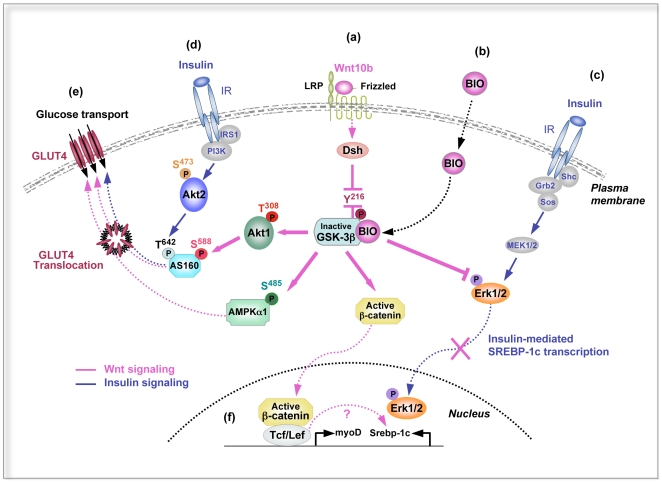
Hypothesis for an interplay between insulin and BIO signaling in contracting myotubes. Wnt10b (**a**) and BIO (**b**) activate the Wnt/β-catenin pathway through the inactivation of GSK-3β^Y216^ phosphorylation, which results in the nuclear translocation of active β-catenin, stimulation of myogenic genes transcription such as myoD, and inhibition of Srebp-1c transcription (**f**). Insulin-induced Srebp-1c transcription is mediated by the MAPK pathway in muscle cells. BIO inhibits the MAPK pathway, which could explain the down-regulation of Srebp-1c gene expression (**c**). In parallel, inactivation of GSK-3β^Y216^ is followed by autophosphorylation of PDK1^S241^ which phosphorylates Akt1^T308^ (but not Akt2^S473^), then the subsequent phosphorylation of AS160^S588^ induces GLUT4 translocation (**e**). In contrast, insulin stimulates GLUT4 translocation through the PI3K/Akt2^S473^/AS160 pathway (**d**). BIO activates the AMP kinase pathway by phosphorylating AMPK-α1^S485^, which also induces GLUT4 translocation (**e**). These results strongly suggest that Wnt signaling, in contrast to insulin signaling, increases glucose transport in both insulin-sensitive and insulin-resistant myotubes through the activation of AMPK-α1 and Akt2/AS160 pathways.

Wnt10b and BIO activate the Wnt/β-catenin pathway through inactivation of GSK-3β^Y216^ both in insulin-sensitive and insulin-resistant myotubes (Figures 8a, 8b), which resulted in nuclear translocation of active β-catenin, stimulation of myogenic genes transcription (e.g myoD, myogenin) and inhibition of Srebp-1c transcription (Figure 8f). In fact, three putative consensus sequences for sterol regulatory elements (SRE) are present in the Wnt10b promoter, and we have already shown negative regulation by Srebp-1c on the mitochondrial uncoupling protein, UCP3, in contracting myotubes [15]. On the other hand, insulin resistance is accompanied by various degrees of impairment of the PI3K signaling [37], whereas the MAPK pathway does not appear defective in the state of insulin resistance [38]. In skeletal muscle cells, insulin-induced Srebp-1c transcription was shown to be mediated by the MAPK pathway, not by the PI3K pathway [39]. In this case, insulin-stimulated expression of SREBP-1c would remain intact in the insulin-resistant state (Fig. 8-c), which could explain increased lipogenesis and intramyocellular lipid deposition. In contrast, stimulation of the Wnt/β-catenin pathway would inhibit the MAPK pathway in insulin-sensitive and insulin-resistant myotubes as well, which could explain the down-regulation of Srebp-1c expression (Figure 8a, 8b). Nevertheless, the mechanism involved in the reciprocal regulation of Wnt10b and Srebp-1c remains to be elucidated at the transcriptional level.

### Hypothesis for an Interplay between Wnt Signaling and Insulin Sensitivity in Muscle Cells

Stimuli that induce GLUT4 translocation in skeletal muscle include insulin via the PI3K pathway [40], hypoxia through nitric oxide signaling [41] and contraction/exercise through AMPK signaling [42]. Wnt signaling stimulated glucose transport independent of insulin, but also restored insulin sensitivity in insulin-resistant myotubes. Inactivation of GSK-3β^Y216^ was followed by the phosphorylation of Akt1^T308^ by PDK1. Then the subsequent phosphorylation of AS160^S588^ induced GLUT4 translocation to the plasma membrane. We don't know yet whether BIO induced PDK1^S241^ autophosphorylation through the inhibition of GSK-3β^Y216^ or via a pathway not yet defined (Figure 8b). In fact, BIO was reported to be an inhibitor of GSK-3α/β through interactions within the ATP binding pocket [30], but also an inhibitor of PDK1 in silico at concentrations ranging from 10 µM to 30 µM [43]. In our study, BIO 1 µM was an activator of PDK1 autophosphorylation in myotubes that were either sensitive or resistant to insulin. Insulin-induced GLUT4 translocation was reported to occur through a pathway involving IRS1/PI3K/PDK2/Akt2^S473^/AS160 in human skeletal muscle [44], and our results show that insulin stimulated a similar pathway in insulin-sensitive myotubes (Figure 8d). In addition, BIO activated the AMPK pathway by phosphorylating AMPK-α1^S485^, but not AMPK-α^T172^ both in insulin-sensitive and insulin-resistant myotubes (Figure 8e). These results are in accordance with those of Jensen et al [29] which found that twitch-contraction increased AMPK-α1 activity, but not AMPK-α2, in mouse skeletal muscle. Altogether our findings demonstrate that Wnt signaling increased glucose transport in both insulin-sensitive and insulin-resistant myotubes through a differential activation of Akt2/AS160 and AMPK-α1 pathways. Nevertheless, how BIO could have a permissive effect on insulin action in insulin-resistant myotubes remains to be determined.

As a switch in the differentiation potential of satellite cells to an adipogenic phenotype could be partly responsible for intramyocellular lipid deposition and insulin resistance in type 2 diabetes, obesity and age-related sarcopenia, organe-selective modulation of the Wnt/β-catenin pathway could contribute to fight intramuscular fat depots and improve insulin sensitivity in these pathologies. For that purpose, the use of GSK-3β-specific inhibitors such as BIO-derivatives may have practical applications in endocrine and regenerative medicine. Nevertheless, as uncontroled canonical Wnt signaling is a hallmark of cancer and other degenerative diseases, understanding the ways in which the pathway is regulated in skeletal muscle is of critical importance.

## Materials and Methods

### Ethics Statement

Animal experiments were conducted in accordance with the Europeen convention STE 123 and the French decree 2001-286.

### Animals and Regeneration Experiments

Regeneration experiments were conducted on crushed Extensor Digitorum Longus (EDL) muscles of 2 month-old Sprague Dawley male rats according to Bassaglia and Gautron [22]. After 2, 8 or 30 days, regenerated muscles were harvested and immediately frozen in liquid nitrogen.

### Primary Culture of Muscle Satellite Cells and Glucose Uptake

Satellite cells from hind limb muscles were isolated and cultured as previously described [45]. Cells were allowed to differentiate onto growth factor-reduced Matrigel™-coated flasks (BD-Biosciences) in DMEM medium containing horse serum, then serum was totally removed to induce contraction. Glucose uptake assay was performed in contracting myotubes using 2-deoxy-D-[1-^3^H]glucose (Amersham Pharmacia Biotech) as previously described [45]. Results are expressed as means±SE. Statistical significance was evaluated using ANOVA.

### RNA Isolation and Semi-Quantitative RT-PCR

Total RNA was extracted using TRIzol reagent (Invitrogen) according to the supplier's instructions. Gene expression was measured using semi-quantitative RT-PCR. Sense and antisense primers against mouse cDNAs were designed as follows: SREBP-1c sense 5′-GGAGCCATGGATTGCACATT-3′; antisense 5′-GCTTCCAGAGAGGAGCCCAG-3′. β-actin sense 5′-TCATGAAGTGTGACGTTGACATCC-3′; antisense 5′-GTAAAACGCAGCTCAGTAACAGTC-3′. MYHC-2 sense 5′GGTACTTGGCGAGAGTGGAG-3′; antisense 5′-AGGGCCAGTGTTTCACATTC-3′; PPAR-β sense 5′-CTCAACATGGAATGTCGGGTGTGC-3′; antisense 5′-CTGATCTCGTTGTAGGGCGGCAGC-3′; PPAR-γ sense 5′-ATGAAGACATTCCATTCACAAGAGC-3′; antisense 5′-ATAGTGGAAGCCAGATGCTTTATCC-3′. Results were obtained from three to four independent RNA samples from individual experiments, each tested in triplicate.

### Preparation of Protein Extracts and Plasma Membranes

Cytoplasmic and nuclear protein extracts were prepared using the NE-PER™ Nuclear and Cytoplasmic Extraction Reagents kit (Pierce) according to the supplier's instructions. Plasma membranes were isolated according to a protocol previously described [15].

### Western Blot Analysis

Cells were immediately frozen in liquid nitrogen, scraped in ice-cold RIPA buffer containing 1 mM PMSF, 2 µg/ml pepstatin A, 2.5 µg/ml benzamidine, 2 µg/ml leupeptin and 5 µg/ml aprotinin, then lysed for 2 hours at 4°C under rotational agitation. Total cell lysates were recovered after centrifugation at 16,000×g for 30 minutes at 4°C to discard insoluble material. Proteins (30 µg) were subjected to immunoblot analysis.

### Antibodies

SREBP-1c was detected using a monoclonal antibody raised against human SREBP-1 (NeoMarkers). Polyclonal antibodies against MyoD, GAPDH, Wnt10b, GLUT4 and β-tubulin were from Santa-Cruz Biotechnology. Rabbit monoclonal antibodies against GSK-3β, Akt, P-Akt2^Ser473^, P-Akt1^Thr308^ and polyclonal antibodies against P-GSK-3β^Ser9^, P-AMPK-α1^Ser485^, P-AMPK-α^Thr172^, P-PDK1^Ser241^ and Lamine A/C were from Cell Signaling. Rabbit monoclonal antibodies against AMPK-α1 and β-catenin were from Epitomics. Monoclonal antibody against active β-catenin was from Chemicon, as well as GLUT1 and AS160 antibodies. Antibody against P-AS160^Ser588^ was from Symansis. Monoclonal P-GSK-3β^Tyr216^ antibody was from BD-Transduction Laboratories. Monoclonal antibodies against MyHC-Dev, MyHC-2 were purchased from Sigma and anti-myogenin from DAKO. Anti-FAS polyclonal antibody was a gift from Dr Dugail (Paris).

### siRNA Design

A target-specific 21-nt siRNA duplex against rat SREBP-1 was designed as previously described [25] and purchased from Dharmacon (Lafayette, CO). Wnt10b siRNA was a pool of 3 target-specific 20-25-nt siRNAs designed to silence mouse Wnt10b gene (Santa-Cruz Biotechnology). Control siRNAs consisted of scrambled sequences that will not lead to the specific degradation of any known cellular mRNA.

### Transfection of Myotubes

Myotubes were cultured in 6-well plates in DMEM without serum, and antibiotics were removed the day before transfection. SiRNA (60 pmoles/well) or pCAGGS plasmid containing the mouse Wnt10b cDNA were transfected using Lipofectamine 2000 (Invitrogen) according to the manufacturer's instructions. Experiments were performed 48 hours later.

### Treatment of Myotubes with 6-Bromo-Indirubin 3′Oxime (BIO)

BIO, a cell-permeable, highly potent, selective, reversible and ATP-competitive specific inhibitor of GSK-3α/β activity, was provided by Drs A. Brivanlou and L. Meijer (The Rockefeller University, New York). Myotubes were treated with 10^−6^ M BIO for 48 hours, or with Me-BIO, an inactive form, as a control, then 2-deoxyglucose uptake was performed, or total protein lysates and plasma membranes were prepared.

### Intramyocellular Lipid Accumulation

Intramyocellular lipids were detected in myotubes using Oil Red O staining according to Koopman et al [46]. Nuclei were counterstained with DAPI (Molecular Probes), then fluorescence was observed using a Nikon TS100 fluorescence microscope.
